# Anonymization-Guided Latent Perturbation for Source-Identity Reduction with Limited Edit Deviation in Instruction-Guided Image Editing

**DOI:** 10.3390/s26196003

**Published:** 2026-09-22

**Authors:** Sanghyeok Seo, Jiwoo Kim, Jong-Uk Hou

**Affiliations:** 1 Division of AI Convergence, Hallym University, Chuncheon 24252, Republic of Korea; a52675268@naver.com; 2 Division of Software, Hallym University, Chuncheon 24252, Republic of Korea; rlawldn0010@gmail.com

**Keywords:** latent diffusion models, generative AI security, identity protection, image editing, adversarial defense, face anonymization, reference-edit deviation

## Abstract

Instruction-guided image editing can modify semantic attributes while retaining the recognizable identity of the source subject, creating risks of unauthorized identity-based manipulation. Existing protection methods can achieve stronger identity disruption, but such outcomes may be accompanied by larger changes to the resulting edit. We investigate a complementary protection strategy and evaluate source-identity reduction jointly with deviation from the corresponding undefended edit. The proposed method constructs an anonymized counterpart of the source image and uses its variational autoencoder (VAE) representation as a source-conditioned latent reference. An $\ell_{\infty }$-bounded pixel-space perturbation is then optimized to move the protected representation toward this anonymization-derived target. Experiments on 9998 CelebA-Wild images show that the proposed method reduces mean face-recognition (FR) similarity from 0.620 to 0.567. In the primary HIVE setting, it achieves the lowest LPIPS and the highest PSNR and SSIM among the evaluated defenses, while FaceLock and the CW-$L_{2}$ attack achieve lower FR similarity at substantially larger reference-edit deviation. On InstructPix2Pix, the proposed method achieves an FR score comparable to PhotoGuard, with an LPIPS of 0.262 versus 0.428 for PhotoGuard. These results indicate that anonymization-guided latent perturbation provides a distinct identity–edit-deviation operating point, reducing source-identity similarity while limiting deviation from the corresponding undefended edit.

## 1. Introduction

Recent advances in latent diffusion models (LDMs) have substantially improved the realism and controllability of image generation and editing [1]. In particular, instruction-guided image-editing methods allow users to modify a given image using natural-language commands while preserving much of the original image structure [2]. Although this capability supports practical applications in creative editing and content generation, it also creates security and privacy risks. A malicious edit can remain recognizable as the original person while altering sensitive attributes such as clothing, expression, background, occupation, or social context. Such identity-preserving manipulation can place a recognizable person in a fabricated semantic context [3].

Adversarial perturbations provide a general mechanism for intentionally changing model behavior with small input modifications [4], while purification methods such as PixelDefend illustrate the complementary idea of mapping perturbed inputs back toward high-probability data regions [5]. In generative image protection, PhotoGuard and EditShield add protective perturbations to interfere with diffusion-based editing [6,7], whereas FaceLock explicitly targets biometric information that remains after malicious editing [8]. These methods optimize different protection objectives and can, therefore, occupy different points between source-identity reduction and deviation from an undefended edit. Accordingly, this work does not treat maximal disruption of the editing process as the only desirable outcome. We instead study whether source-identity similarity can be reduced while limiting the amount by which the edited output departs from the corresponding undefended edit.

Motivated by this perspective, we investigate an identity-oriented protection method where behavior is evaluated jointly in terms of source-identity similarity and deviation from the corresponding undefended edit. As illustrated in Figure 1, the proposed method uses an anonymized counterpart of the same source image as a source-conditioned reference in the VAE latent space. A small pixel-space perturbation is optimized so that the protected image representation approaches this reference. This construction does not assume that Euclidean distance in the VAE latent space is identity-specific, nor does it imply that the final edited output reproduces the anonymized target identity. To examine whether the choice of optimization target affects the observed trade-off, we additionally compare the proposed source-matched anonymization target with random-image and grayscale-image targets under the same optimization setting. We also directly measure the FR similarity between the anonymized target and the protected edited output to test whether the final edit simply inherits the anonymized counterpart’s identity.

As illustrated in Figure 2, we evaluate source-identity reduction together with the amount of change introduced relative to the corresponding undefended edit. The proposed method does not achieve the lowest FR similarity among all evaluated defenses. Instead, its main empirical characteristic in the primary setting is that the reduction in mean FR similarity from 0.620 to 0.567 is accompanied by the highest PSNR and SSIM and the lowest LPIPS among the evaluated defenses. A similar pattern is observed on InstructPix2Pix (IP2P), where the proposed method obtains an FR score nearly identical to PhotoGuard while showing substantially lower LPIPS. We, therefore, interpret the method as occupying a different empirical identity–edit-deviation operating point rather than as universally outperforming stronger disruption-oriented or identity-oriented defenses. The main contributions of this work are as follows:

- We evaluate identity-oriented protection for instruction-guided image editing by jointly considering source-identity reduction and deviation from the corresponding undefended edit.
- We propose a source-conditioned target construction in which an anonymized counterpart of the input provides the VAE-latent reference for a bounded pixel-space perturbation. Target-directed latent optimization itself is not claimed as new; the design distinction is the use of an anonymization-derived counterpart of the same source image as the reference.
- We evaluate the approach on 9998 images. In the primary setting, the proposed method provides moderate source-identity reduction while obtaining the highest PSNR and SSIM and the lowest LPIPS among the evaluated defenses, indicating a smaller deviation from the corresponding undefended edits under these metrics.
- Through loss-component, target-construction, perturbation-budget, threshold-based face-verification, and practical-preprocessing analyses, we characterize the observed trade-off within the tested formulation. We additionally report an IP2P evaluation and qualitative observations on HIVE, IP2P, and DreamBooth, while avoiding claims of comprehensive cross-model robustness or target-identity replacement.

**Figure 2:**
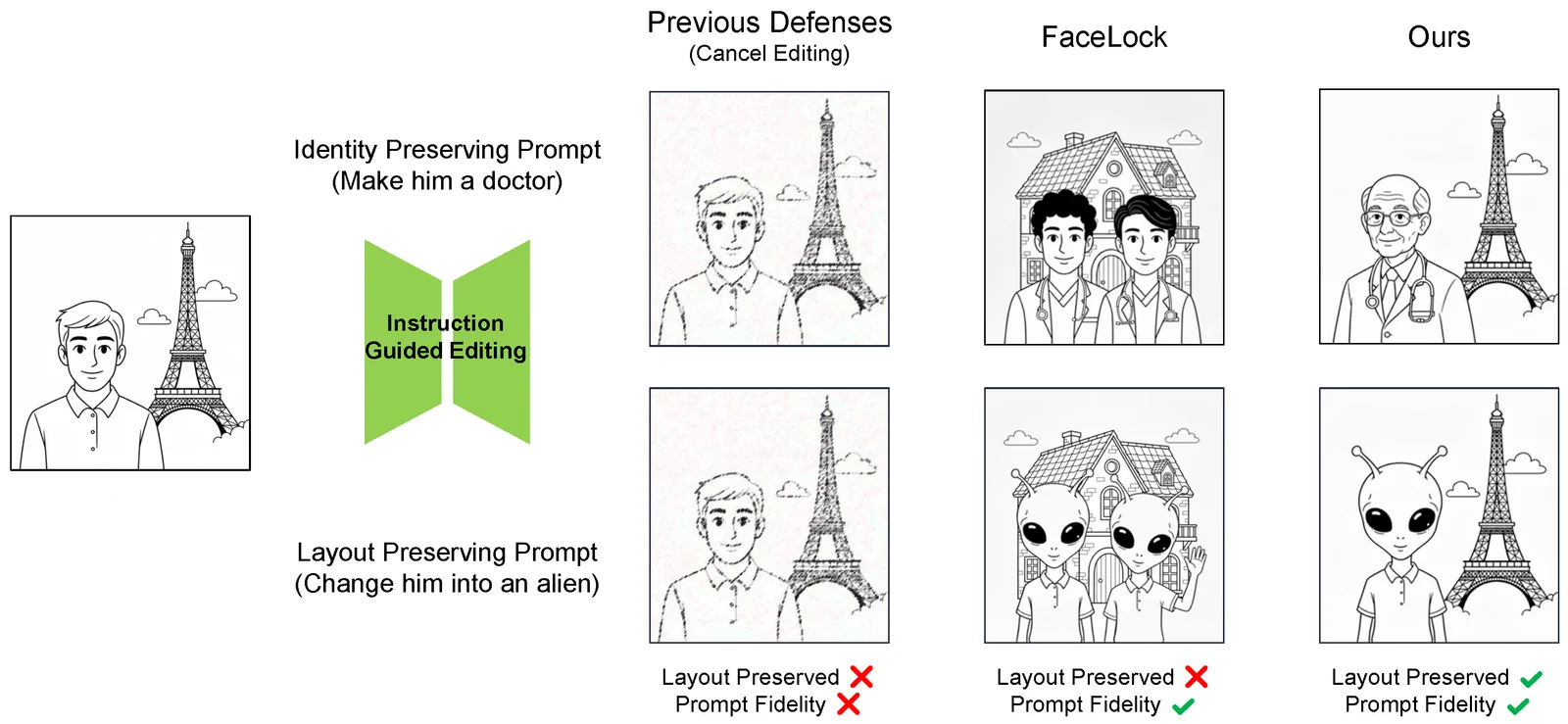
Conceptual comparison of protection behavior in terms of source-identity reduction and deviation from the corresponding undefended edit. The disruption-oriented column is schematic and is not intended to characterize every prior defense. FaceLock represents an identity-oriented baseline, while the proposed method is evaluated along both axes. This illustration represents the intended evaluation perspective and is not used as quantitative evidence of contextual-attribute or layout preservation.

## 2. Related Work

### 2.1. Instruction-Guided Image Editing and Identity-Preserving Misuse

LDMs perform the diffusion process in a compact latent representation, enabling efficient high-quality image generation and editing [1]. Instruction-based editing methods such as InstructPix2Pix demonstrated that text-guided edits can be learned from automatically generated editing pairs [2]. Prompt-to-Prompt controls text-driven modifications through cross-attention manipulation [9], whereas Pix2Pix-Zero performs zero-shot image-to-image translation with pretrained text-to-image diffusion models while using cross-attention guidance to preserve source structure [10]. More recent approaches, such as HIVE, incorporate human feedback to improve instructional visual editing [11]. The ability of these systems to preserve recognizable source content while modifying requested semantics is valuable for benign editing but can also facilitate unauthorized identity-preserving manipulation. Consequently, the threat considered in this work is not image modification itself, but manipulation in which the source subject remains recognizable after semantic editing.

### 2.2. Adversarial Defenses Against Generative Editing

Adversarial machine learning has established that small input perturbations can substantially alter model behavior [4]. Purification-based defenses such as PixelDefend approach the inverse problem by moving adversarial inputs toward regions of higher data likelihood [5]. In the broader generative-AI protection literature, Glaze protects artists against style mimicry and Nightshade targets unauthorized model training through prompt-specific poisoning [12,13]. These objectives differ from protection against instruction-guided image editing. For malicious image editing, PhotoGuard introduces image immunization strategies including an encoder attack that steers the encoder output toward a target latent representation [6]. EditShield similarly perturbs representations used by instruction-guided diffusion models so that subsequent edits can become mismatched or unrealistic [7]. FaceLock is more directly related to the threat studied here because it explicitly targets biometric information using facial-recognition and visual-perception objectives [8]. The proposed method does not claim novelty for target-directed latent optimization itself. Its design difference is the construction of a source-conditioned reference by anonymizing the same source image and using the resulting VAE representation as the target. We, therefore, include a matched target-construction control in which random-image, grayscale-image, and source-matched anonymization targets are compared under the same optimization setting. This control is used to assess whether the proposed target provides a favorable identity–edit-deviation operating point within the tested alternatives; it is not interpreted as proof that the anonymization-derived target is universally optimal.

### 2.3. Relation to Face Anonymization

Face anonymization aims to alter or remove identifiable facial information while retaining useful non-identity content. Traditional approaches may blur, mask, or replace facial regions, whereas recent generative approaches can synthesize more realistic anonymized faces. We use the diffusion-based anonymization method of Kung et al. [14] to construct an anonymized counterpart of each source image. Their method is designed to alter facial identity while preserving facial attributes and image quality; in our framework, however, the anonymized image is not released as the protected output and is not treated as a guaranteed target identity. Instead, it provides a source-conditioned latent reference for optimizing a small perturbation applied to the original image. The present study evaluates the downstream behavior of this construction through source-identity and edit-deviation metrics.

## 3. Joint Evaluation of Identity Reduction and Edit Deviation

Protection methods for generative editing need not be compared along a single axis. A method can reduce the recognizability of the source subject strongly while also causing large changes to the resulting edit, whereas another method can produce a smaller identity shift together with a smaller departure from the undefended edit. We, therefore, analyze source-identity reduction jointly with edit deviation. Within this perspective, the proposed approach investigates a source-conditioned target derived from an anonymized version of the same input. Specifically, the latent representation of the protected image is optimized toward the VAE representation of the anonymized counterpart. This target is chosen because the anonymization procedure is intended to alter facial identity while retaining other visual content, but the VAE latent space itself is not assumed to provide an explicit decomposition of identity and non-identity factors. Consequently, proximity to the anonymized latent target is an operational optimization objective rather than a semantic guarantee. The target-construction control reported in Section 5.7 compares this reference with random-image and grayscale-image targets, while Section 5.8 directly measures the similarity between the protected edit and anonymized target. These experiments support the tested operating point without implying universal optimality or explicit identity replacement. Throughout this paper, we, therefore, describe the observed behavior as source-identity reduction, identity disruption, identity alteration, or an anonymization-guided latent shift rather than explicit identity replacement.

## 4. Materials and Methods

### 4.1. Anonymization-Guided Latent Perturbation

The complete procedure is illustrated in Figure 1. Given an original image *x*, we first generate an anonymized counterpart $x_{\mathrm{anon}}$ using the method of Kung et al. [14]. In the proposed framework, the anonymized image is not treated as the protected output itself. Instead, it provides a source-conditioned reference for latent-space optimization. Specifically, the anonymized image is encoded using the VAE encoder *E* to obtain

(1) $$z_{anon}=E\left(x_{anon}\right).$$

For a pixel-space perturbation δ, the latent representation of the perturbed image is

(2) $$z_{perturbed}=E\left(x+\delta \right).$$

The primary objective, referred to as the anonymization proximity loss $L_{\mathrm{anon}}$, is

(3) $$L_{anon}={∥z_{perturbed}-z_{anon}∥}_{2}^{2}.$$

The complete optimization problem is

(4) $$\underset{\delta }{argmin}\;{∥E\left(x+\delta \right)-z_{anon}∥}_{2}^{2}\;s.t.\;{∥\delta ∥}_{\infty }\leq \epsilon .$$

The protected image is subsequently defined as

(5) $$x_{\text{protected}}=x+\delta .$$

The $\ell_{\infty }$ constraint restricts the maximum pixel-wise perturbation magnitude and, therefore, bounds the change applied to the source image in pixel space. We do not interpret this constraint alone as a guarantee of human perceptual imperceptibility. Likewise, the VAE-latent objective does not explicitly isolate identity dimensions from pose, expression, background, or other content. Its role is to bias the protected representation toward the anonymization-derived reference before downstream instruction-guided editing, after which the effect is assessed empirically using the reported metrics.

### 4.2. Auxiliary Losses for Ablation

The default proposed method uses only the anonymization proximity loss $L_{\mathrm{anon}}$ in Equation (3). We additionally examine whether explicitly adding stronger representation- or identity-oriented objectives improves the observed identity–edit-deviation trade-off. For this purpose, two auxiliary terms are evaluated only in the loss-component ablation: $L_{\mathrm{disrupt}}$, an additional representation-disruption term, and $L_{\mathrm{FR}}$, an additional face-recognition-based term. These auxiliary terms are not part of the default formulation used for the main results. For the ablation experiments, the optimization objective can be written generically as

(6) $$L_{abl}=L_{anon}+\lambda_{d}L_{disrupt}+\lambda_{f}L_{FR},$$

where $\lambda_{d}$ and $\lambda_{f}$ control whether the corresponding auxiliary terms are included. The default method corresponds to excluding both auxiliary terms. The purpose of this experiment is not to redefine the proposed objective, but to test whether adding explicit disruption-oriented signals provides a more favorable balance between source-identity reduction and deviation from the corresponding undefended edit.

### 4.3. Experimental Setup

#### 4.3.1. Models and Dataset

HIVE [11] is used as the primary instruction-guided editing model. The proposed perturbation is optimized through the VAE encoder used in the Stable Diffusion/LDM pipeline [1]. The anonymized counterpart $x_{anon}$ is generated using the method of Kung et al. [14]. We evaluate 9998 images from CelebA-Wild. The initial subset contained 10,000 images, but two images were excluded because face detection failed, making them unsuitable for the identity evaluation. The editing prompts follow the categories used in FaceLock and include profession changes, background modifications, and accessory additions [8].

#### 4.3.2. Baselines

The evaluated baselines include PhotoGuard [6], EditShield [7], FaceLock [8], an untargeted encoder attack, a CW-$L_{2}$ attack [15], and a VAE attack. These baselines span both broad editing-disruption objectives and identity-oriented protection, and they are, therefore, interpreted according to the joint identity–edit-deviation results rather than as interchangeable instances of a single defense objective.

#### 4.3.3. Evaluation Metrics

We evaluate source-identity similarity, source-to-edit visual similarity, deviation from the corresponding undefended edit, and prompt-related editing behavior from complementary perspectives.

- **Identity similarity:** Following the evaluation protocol of FaceLock [8], FR similarity is computed between the source and edited images using CVLFace, specifically the AdaFace ViT-Base model with keypoint-relative position encoding trained on WebFace4M. Faces are aligned using the CVLFace DFA MobileNet aligner (CVLFace, https://github.com/mk-minchul/CVLface, accessed on 19 September 2026). FR similarity is then computed as the cosine similarity between the resulting face embeddings. In the FaceLock evaluation pipeline used for Table 1, the alignment confidence is not used as an additional threshold for excluding source–edit pairs; therefore, the FR values in Table 1 are not computed from the detection-success counts reported separately in the face-detectability analysis. Table 1 is computed over the common evaluation population of 9998 source–edit pairs; the separate detection counts from the face-detectability analysis are not used to construct method-specific FR subsets. A lower FR score indicates lower similarity to the source identity.
- **Source-to-edit visual similarity:** Following the FaceLock evaluation protocol [8], CLIP image similarity (CLIP-I) is computed as the cosine similarity between the source and edited image embeddings using OpenAI CLIP ViT-B/32 (OpenAI CLIP, https://github.com/openai/CLIP, accessed on 19 September 2026) [16]. Higher values indicate greater source-to-edit visual similarity.
- **Reference-edit deviation:** PSNR, SSIM [17], and LPIPS [18] compare the edited output produced from the protected input with the corresponding edited output produced from the unprotected input. Higher PSNR and SSIM and lower LPIPS indicate smaller deviation from that reference edit. These metrics are not interpreted as direct measurements of human perceptual quality.
- **Prompt-related score:** CLIP-S is computed following the FaceLock evaluation protocol [8] using OpenAI CLIP ViT-B/32 [16]. Specifically, it measures the cosine similarity between the source-to-edit image-embedding shift and the editing-instruction text embedding, thereby quantifying prompt-consistent editing behavior.

For the supplementary threshold-based verification analysis, we calibrate cosine-similarity thresholds using source–source impostor pairs. The calibration set contains 200 source images corresponding to distinct identities. Among these, 125 source images with valid face embeddings yield 7750 cross-identity impostor pairs. The resulting thresholds are 0.3598 at FAR = 1% and 0.6325 at FAR = 0.1%. These fixed thresholds are then applied to the same common set of 432 valid source–edit pairs for both the undefended HIVE outputs and the proposed method.

These metrics are interpreted jointly because a low FR score can be obtained together with substantial changes to the edited output. The present study reports descriptive means and standard deviations where available and does not make claims of statistical significance.

#### 4.3.4. Implementation Details

The perturbation budget is set to ϵ=0.02, following the setting used in FaceLock [8]. For a fair comparison, all evaluated protection methods—PhotoGuard, EditShield, FaceLock, the untargeted encoder attack, the CW-$L_{2}$ attack, the VAE attack, and the proposed method—were run with the same $\ell_{\infty }$ perturbation budget of ϵ=0.02 in our experiments. Optimization of the proposed method is performed for 100 iterations using MI-FGSM [19]. The momentum decay factor is set to μ=1.0, and the gradient at each iteration is normalized by its $L_{1}$ norm before accumulation in the momentum term. Because image tensors are represented in the [−1,1] range during optimization, ϵ=0.02 in the original [0,1] image scale corresponds to a maximum perturbation magnitude of 0.04 in the optimization space. The initial step size is set to 0.04 and is linearly decayed over the 100 optimization iterations. The perturbation-optimization stage takes approximately 7.3 s per image on a single NVIDIA RTX 4090 GPU (NVIDIA Corporation, Santa Clara, CA, USA). With the anonymization model already loaded, the anonymized-target generation stage averages approximately 16.7 s per image in our implementation. The sequential end-to-end protection cost is, therefore, approximately 24.0 s per image, excluding downstream image editing; at this rate, processing 10,000 images requires approximately 66.7 h (about 2.8 days). Non-square CelebA-Wild images are padded to a square format during optimization and subsequently restored to their original aspect ratio by removing the padding. The proposed perturbation is, therefore, described as a pixel-space perturbation optimized through a latent-space objective.

## 5. Results

### 5.1. Quantitative Identity–Edit-Deviation Trade-Off

Table 1 reports source-identity similarity, prompt-related behavior, source-to-edit visual similarity, and reference-edit deviation. The proposed method reduces mean FR similarity from 0.620 in the unprotected setting to 0.567, indicating a lower average similarity to the source identity. However, the proposed method does not achieve the lowest FR value among the evaluated defenses: FaceLock and the CW-$L_{2}$ attack achieve lower FR scores of 0.496 and 0.516, respectively. The main empirical difference is observed in the metrics that compare defended edits with their corresponding undefended edits. The proposed method achieves an LPIPS value of 0.278, compared with 0.431 for FaceLock and 0.450 for the CW-$L_{2}$ attack, together with the highest PSNR of 22.93 and the highest SSIM of 0.747 among the evaluated defense methods. We, therefore, interpret the result as an identity–edit-deviation operating point under the reported metrics rather than as evidence of the strongest identity disruption or direct human-perceptual quality superiority.

### 5.2. Qualitative Results

Figure 3 presents the representative results obtained using different protection methods and editing instructions.

The examples are intended to complement the quantitative identity–edit-deviation analysis rather than to independently establish preservation of particular attributes.

As shown in Figure 3, several baselines that achieve stronger source-identity reduction also produce visibly larger changes in some examples. In contrast, the proposed method often retains recognizable editing behavior while exhibiting facial changes. This observation is consistent with Table 1, where the proposed method does not obtain the lowest FR score but shows smaller reference-edit deviation, according to LPIPS, PSNR, and SSIM, than several stronger identity-disruption baselines. Figure 3 is interpreted only as a qualitative complement to the quantitative results; the current experiments do not independently quantify preservation of pose, expression, background, or layout.

### 5.3. Qualitative Observations Across Editing Models

We additionally examine the protected inputs using multiple generative editing pipelines. Figure 4 presents representative examples from HIVE, InstructPix2Pix, and DreamBooth.

The examples in Figure 4 suggest that the observed facial changes are not visually restricted to the primary HIVE editor. Similar qualitative behavior can also be observed in the examples generated by IP2P and DreamBooth. However, this experiment does not provide a quantitative transfer matrix across editing models. We, therefore, interpret the examples as exploratory cross-editor observations rather than evidence of broad or model-independent robustness. A systematic quantitative analysis across different editing models, VAEs, and face-recognition models remains as future work.

### 5.4. Threshold-Based Face-Verification Analysis

Mean FR cosine similarity provides a continuous measure of source–edit identity similarity, but it does not directly indicate how often an edited output would still be accepted as the same identity at a fixed verification operating point. We, therefore, additionally evaluate the true acceptance rate (TAR) at fixed false acceptance rates (FARs). The calibration set contains 200 source images corresponding to distinct identities. Among these, 125 source images with valid face embeddings produce all 1252=7750 cross-identity impostor pairs. From this impostor distribution, the cosine-similarity thresholds corresponding to FAR = 1% and FAR = 0.1% are 0.3598 and 0.6325, respectively. The empirical FARs at these thresholds are 0.010065 and 0.001032.

For a direct comparison, we use the same common subset of 432 valid source–edit pairs for the undefended HIVE outputs and the proposed method. Because this common subset differs from the full population used in Table 1, the mean FR values in this analysis are not expected to match the full-dataset means. The results are summarized in Table 2.

At FAR = 1%, TAR decreases from 70.60% for the undefended HIVE outputs to 68.29% for the proposed method, an absolute reduction of 2.31 percentage points. At FAR = 0.1%, TAR decreases from 53.47% to 47.92%, corresponding to an absolute reduction of 5.55 percentage points (approximately 10.39% relative). These results show that the observed reduction in mean FR similarity is accompanied by a measurable reduction in same-identity acceptance at fixed FAR operating points.

### 5.5. Face-Detectability Analysis

A low FR score alone is insufficient to distinguish identity alteration from severe facial corruption. We, therefore, report the available face-detector outputs as a limited descriptive proxy. These detector outputs are analytically separate from the FR population used in Table 1; the detection counts in Table 3 are not used to filter the source–edit pairs for the main FR calculation. As shown in Table 3, the proposed method obtains an average detection confidence of 0.6447, close to the 0.6455 reported in the unprotected condition. The detection counts are reported exactly as produced by the evaluation procedure, but they are not normalized here into a full-dataset detection rate and are, therefore, not interpreted as calibrated detection probabilities. This analysis only indicates that the reported detector confidence for the proposed method is similar to that of the unprotected condition; it does not establish comprehensive facial structural integrity, perceptual naturalness, or successful identity protection.

### 5.6. Loss-Component Analysis

Table 4 examines whether the two auxiliary losses introduced in Equation (6) improve the observed identity–edit-deviation trade-off. These terms are evaluated only as ablation variants; the default proposed method uses $L_{anon}$ alone. With $L_{anon}$ alone, the method obtains an FR score of 0.567 and an LPIPS value of 0.278. Adding $L_{disrupt}$ reduces FR to 0.547 but increases LPIPS to 0.321, while adding $L_{FR}$ gives an FR score of 0.551 with the same LPIPS value of 0.321. Activating both auxiliary terms yields an FR score of 0.547 and an LPIPS value of 0.322. Thus, the additional disruption-oriented objectives provide only a modest further reduction in source-identity similarity while increasing deviation from the corresponding undefended edit. This result motivates the use of $L_{anon}$ alone as the default objective for the main experiments. Importantly, the ablation concerns loss-component combinations rather than target construction: it does not establish that the anonymization-derived target is superior to gray, random, or other possible targets, nor does it show that the final edited output matches the anonymized target identity.

### 5.7. Target-Construction Control Experiment

To examine whether the optimization target itself affects the identity–edit-deviation trade-off, we repeat the protection procedure under the same optimization setting while changing only the latent-space target. We compare a random-image target, a grayscale-image target, and the proposed source-matched anonymization target. Table 5 reports FR similarity between the source and protected edit together with MSE and SSIM measured against the corresponding undefended HIVE edit.

The random-image target produces the strongest reduction in source-identity similarity, with an FR score of 0.4191, but it also causes the largest deviation from the undefended edit, as reflected by an MSE of 0.0210 and an SSIM of 0.8183. The grayscale and source-matched anonymization targets yield similar FR scores (0.4860 and 0.4889, respectively). However, the source-matched anonymization target produces a lower MSE (0.0093 versus 0.0101) and a higher SSIM (0.9196 versus 0.9127). Within these tested controls, the proposed target, therefore, does not maximize identity suppression; rather, it provides a more favorable balance between source-identity reduction and preservation of the corresponding undefended edit than the random target, and slightly smaller edit deviation than the grayscale target at a similar FR level.

### 5.8. Similarity to the Anonymized Target

Because the anonymized counterpart is used as the latent optimization target, we additionally test whether the protected edited output directly inherits the identity of that target. Using the same face-recognition model as in the main evaluation, we compute pairwise cosine similarities among the source image, anonymized target, normal HIVE edit, and protected HIVE edit. The results are shown in Table 6.

The anonymized target–protected edit similarity is 0.3230, which is lower than the source–protected edit similarity of 0.4889. Thus, although the anonymized counterpart guides the latent optimization, the final edited output does not simply reproduce or inherit the anonymized target identity according to the evaluated FR embedding. The result supports interpreting the method as an anonymization-guided reduction in source-identity similarity rather than explicit identity replacement.

### 5.9. Evaluation on InstructPix2Pix

We additionally evaluate the proposed method on InstructPix2Pix to examine its behavior on an editing model different from the primary HIVE setting. Table 7 reports FR similarity and LPIPS. The proposed method achieves an FR score of 0.544, nearly identical to PhotoGuard at 0.543 and lower than the unprotected baseline at 0.610. Several baselines, including the encoder attack, CW attack, and FaceLock, achieve substantially lower FR values; therefore, the IP2P experiment is not interpreted as showing state-of-the-art identity disruption. In contrast, the proposed method achieves the lowest LPIPS value (0.262) among the evaluated defenses. PhotoGuard, EditShield, and FaceLock obtain LPIPS values of 0.428, 0.351, and 0.477, respectively. Thus, the main observation on IP2P is that a moderate decrease in source-identity similarity is accompanied by a smaller deviation from the corresponding undefended edit under LPIPS. This pattern is consistent with the primary HIVE evaluation, although it does not establish broad cross-model robustness.

### 5.10. Perturbation-Budget Analysis

Table 8 summarizes the effect of the perturbation budget ϵ. As ϵ increases from 0.01 to 0.05, FR similarity decreases consistently from 0.610 to 0.487, indicating greater source-identity reduction at larger perturbation budgets. LPIPS, however, changes non-monotonically: it increases sharply at ϵ=0.03, decreases at ϵ=0.04, and remains higher than the values observed at the two smallest budgets. The present experiment does not identify whether this non-monotonic behavior is caused by seed-dependent stochasticity, prompt-specific effects, or sample-level variation; we, therefore, report it descriptively rather than attributing it to a specific mechanism. Importantly, ϵ=0.02 was adopted a priori following the FaceLock setting and was not selected post hoc from this perturbation-budget sweep. A systematic multi-seed evaluation across editing prompts, together with sample-level outlier analysis, is left for future work to assess the stability and origin of this non-monotonic behavior.

### 5.11. Robustness to Practical Image Preprocessing

To examine whether the perturbation-based protection remains effective after common image preprocessing, we apply JPEG compression, median filtering, resizing, and center cropping to the protected inputs before HIVE editing. Each condition contains the same 800 edited outputs (200 source images × 4 prompts). For a consistent reference, the clean protected condition is recomputed on exactly the same 800-image subset rather than reusing the full-dataset result. The results are summarized in Table 9.

In the clean protected condition, the mean FR similarity is 0.4889. After preprocessing, FR remains close to this value: 0.4854 under JPEG Q = 50, 0.5010 under JPEG Q = 75, 0.5005 after 3×3 median filtering, 0.4890 after 0.75× resizing, and 0.4824 after 0.90 center cropping. The largest increase relative to the clean protected condition is approximately 0.012, observed for JPEG Q = 75. These results indicate that the source-identity suppression effect is largely retained under the tested preprocessing operations, although the magnitude varies slightly across transformations.

LPIPS, PSNR, and SSIM are additionally reported to characterize changes in the resulting edited outputs. JPEG compression, resizing, and median filtering remain relatively close to the clean condition, whereas center cropping produces a substantially larger deviation because it directly changes the spatial content of the input. Importantly, the perceptual and structural metrics under preprocessing reflect both the effect of the defense and the change introduced by the preprocessing operation itself. Because an identically preprocessed undefended branch was not separately evaluated, these metrics should not be interpreted as isolating the defense-specific contribution. We, therefore, interpret the results as evidence of partial robustness to the tested preprocessing operations rather than complete preprocessing invariance.

## 6. Discussion

The experiments support a narrow but consistent empirical conclusion: under the evaluated settings, the proposed method provides moderate source-identity reduction while introducing smaller deviation from the corresponding undefended edits than several stronger identity-disruption baselines, according to LPIPS, PSNR, and SSIM. FaceLock and CW-$L_{2}$ achieve lower FR values in the primary HIVE setting, and several methods also achieve stronger identity reduction on IP2P; therefore, we characterize the proposed method by its identity–edit-deviation operating point rather than by strongest-defense or universal-superiority claims. The threshold-based verification analysis further shows that the decrease in mean FR is accompanied by lower same-identity acceptance, particularly at the stricter FAR operating point, without implying that identity verification is completely defeated.

The additional analyses help clarify the role of the proposed formulation. The random-image target yields stronger identity suppression but substantially larger edit-output deviation, whereas the source-matched anonymization and grayscale targets achieve similar FR values and the proposed target produces slightly smaller deviation. Moreover, the anonymized-target–protected-edit FR similarity is lower than the source–protected-edit similarity, providing no evidence that the final edit simply copies the anonymized target identity. The loss-component ablation leads to a similar conclusion: adding the tested disruption- or FR-oriented auxiliary terms slightly lowers FR but increases LPIPS, and, therefore, does not improve the measured trade-off under the present setting.

Several limitations constrain the interpretation of these results. The $\ell_{\infty }$ constraint bounds the maximum pixel-wise perturbation but does not itself establish human perceptual imperceptibility. Likewise, FR cosine similarity is an embedding-space proxy for identity similarity, while LPIPS, PSNR, and SSIM do not fully capture facial naturalness, prompt consistency, semantic variation, or subjective identity perception. Because no human-subject evaluation was conducted, these metrics should be interpreted as objective proxies rather than direct measures of subjective perceptual quality. Future work should, therefore, include human evaluation of perceived identity similarity, editing naturalness, and instruction consistency.

Robustness and generalization are also limited by the present experimental scope. The HIVE, IP2P, and DreamBooth comparison is partly qualitative, and the current experiments do not separately quantify preservation of pose, expression, background, or layout. The practical-preprocessing study shows that source-identity reduction remains observable after the tested JPEG compression, median filtering, resizing, and center cropping, but only a limited set of transformations and severity levels was evaluated on HIVE. Because the undefended reference branch was not subjected to identical preprocessing, LPIPS, PSNR, and SSIM, under these transformations, combine preprocessing-induced and defense-induced changes and should not be interpreted as isolating the defense-specific contribution. Broader purification procedures, repeated preprocessing, VAE re-encoding, and additional editing models remain to be studied. Sensitivity to the external anonymization model has also not yet been quantified; accordingly, we do not claim anonymization-model-independent robustness, and evaluation with alternative face-anonymization models and cross-anonymizer sensitivity analysis remain important future directions. The face-detection results are descriptive rather than calibrated full-dataset detection-rate measurements, and the reported means and standard deviations are not interpreted as evidence of statistical significance.

The method also incurs a nontrivial computational cost because anonymized-target generation precedes perturbation optimization. In our implementation, the complete model-loaded sequential protection pipeline requires approximately 24.0 s/image. For the literature context, previously reported protection-generation runtimes are approximately 2.46 s/image for EditShield on an RTX 4090 [7], approximately 20 s/image for FaceLock when the FR model is included [8], and 207.0 s/image for PhotoGuard-E in the DiffVax benchmark [20]. Because these values were obtained using different hardware, implementations, and settings, they should not be treated as a controlled head-to-head efficiency comparison. Overall, the current evidence supports the reported empirical operating point, but not stronger claims of universal robustness, human-perceptual superiority, target-identity replacement, or superior computational efficiency.

## 7. Conclusions

We investigated an anonymization-guided latent perturbation strategy for reducing source-identity similarity in instruction-guided image editing. The method uses the VAE representation of an anonymized counterpart of the same source image as the target of an $\ell_{\infty }$-bounded pixel-space perturbation. The experiments show that the proposed method is not the strongest identity-disruption approach among the evaluated defenses. Instead, it provides moderate source-identity reduction together with comparatively small deviation from the corresponding undefended edits according to the reported LPIPS, PSNR, and SSIM metrics. In the primary evaluation, the proposed method obtains the highest PSNR and SSIM and the lowest LPIPS among the evaluated defenses. On IP2P, its FR score is comparable to PhotoGuard while its LPIPS is lower.

These findings are interpreted as an empirical identity–edit-deviation operating point under the tested settings. The threshold-based verification analysis further shows that the FR reduction is accompanied by lower TAR at fixed FAR operating points, including a decrease from 53.47% to 47.92% at FAR = 0.1%. The target-construction control shows that the random target achieves stronger identity suppression at substantially larger edit deviation, while the source-matched anonymization target yields slightly smaller deviation than the grayscale target at a similar FR level. The low FR similarity between the anonymized target and the protected edit further indicates that the final edited output does not simply inherit the anonymized counterpart’s identity. In addition, the practical-preprocessing experiment shows that the source-identity reduction effect remains observable after the tested JPEG compression, median filtering, resizing, and center-cropping operations, although the magnitude of the effect and edit deviation vary across transformations. These results do not establish the universal optimality of the proposed target, direct human-perceptual quality superiority, or robustness across all editing models and practical transformations. They, nevertheless, show that identity-oriented image protection can be evaluated meaningfully by considering source-identity reduction together with the amount of change introduced into the resulting edit.

## Figures and Tables

**Figure 1:**
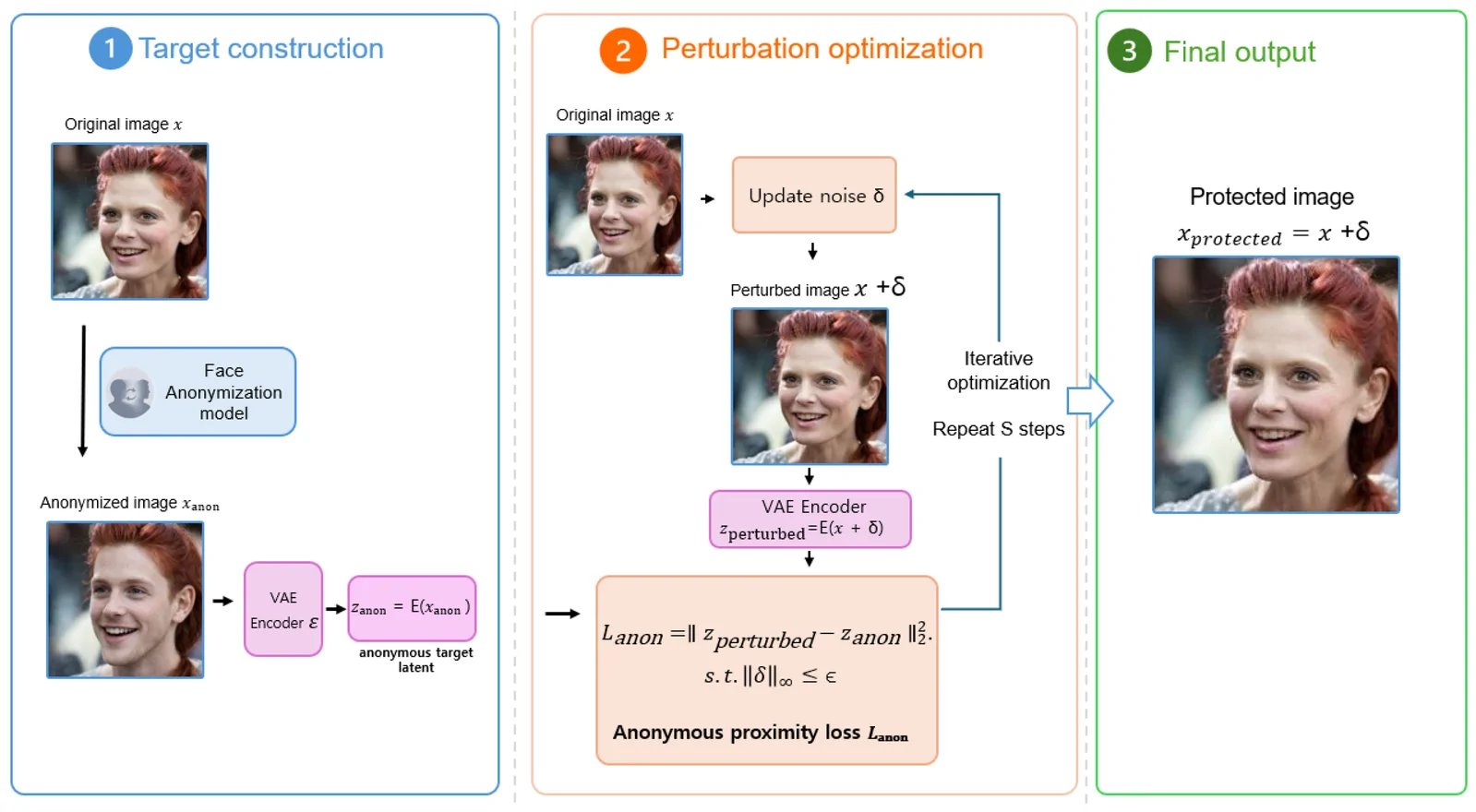
Overall pipeline of the proposed anonymization-guided identity-protection method. Given an original image *x*, an anonymized counterpart $x_{\mathrm{anon}}$ is first generated and encoded into the VAE latent space. A small pixel-space perturbation δ is subsequently optimized so that the VAE representation of the protected image approaches the latent representation of the anonymized counterpart. The resulting protected image is then provided to an instruction-guided editing model. The anonymized counterpart is used only as a source-conditioned latent optimization reference; the formulation does not guarantee that the final edited output matches a specific anonymous identity.

**Figure 3:**
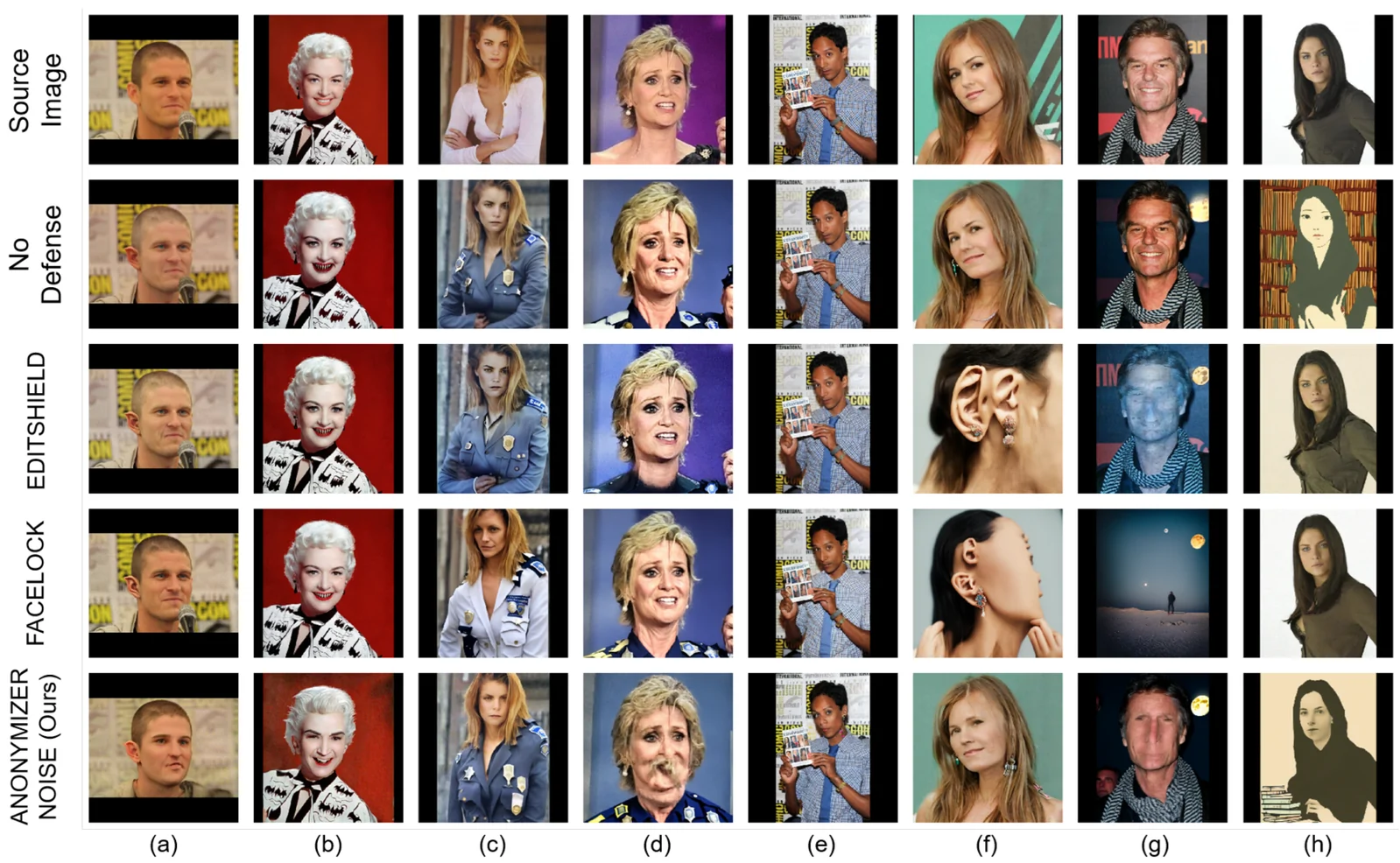
Qualitative comparison of different defense methods across multiple instruction-guided editing tasks. The eight columns correspond to distinct source-image/editing-instruction pairs: (**a**) a facial-appearance editing example; (**b**) an example corresponding to “Add large vampire fangs”; (**c**) another appearance-related editing example; **(d**) an example corresponding to “Let the person wear a police suit”; (**e**) an additional appearance-related editing example; (**f**) an example corresponding to “Let the person wear earrings”; (**g**) an example corresponding to “Let the person stand under the moon”; and (**h**) an example corresponding to “Set the background in a library”.

**Figure 4:**
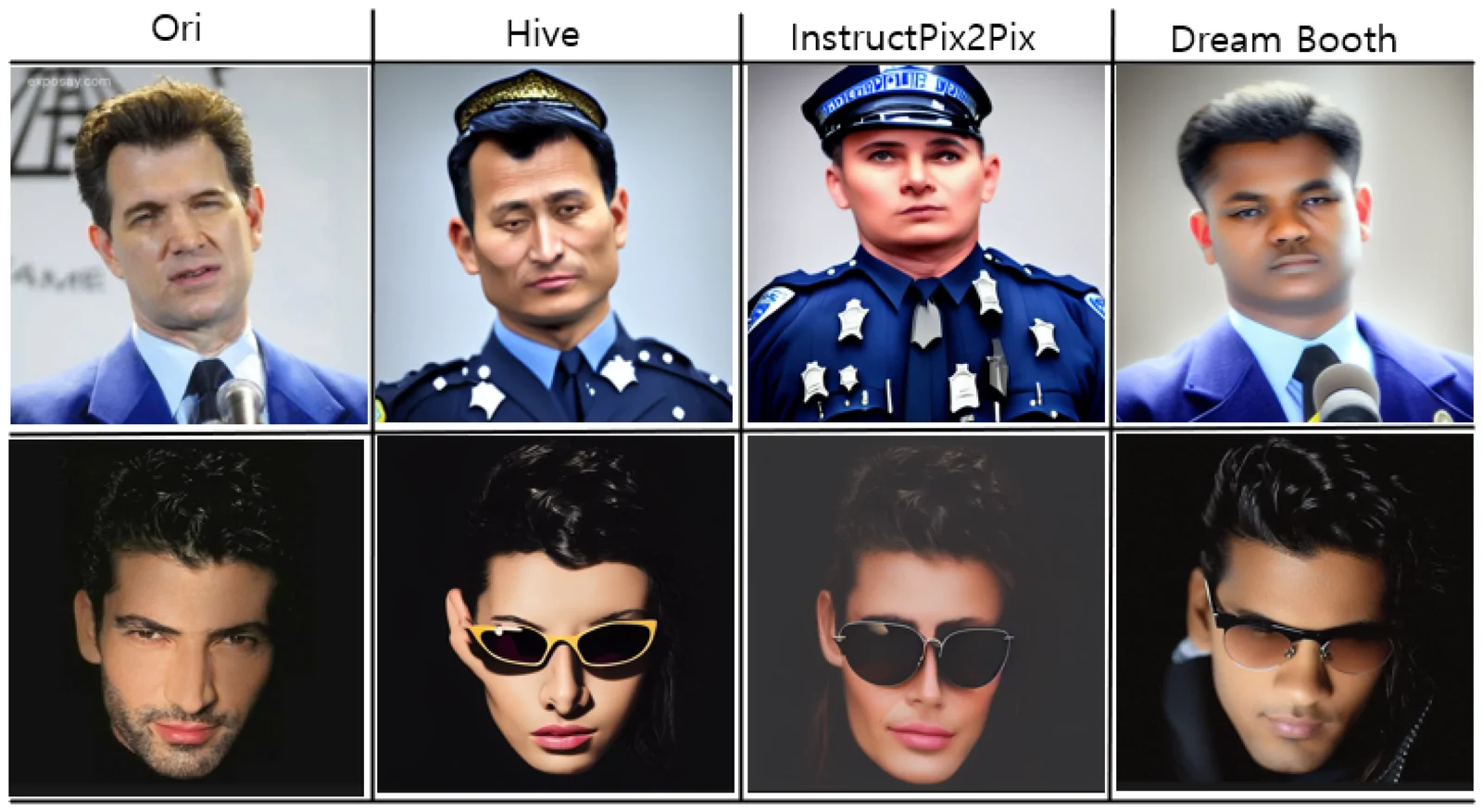
Qualitative observations across different generative editing models, including HIVE, InstructPix2Pix, and DreamBooth. Changes in facial appearance can be visually observed in examples from multiple editing pipelines while the requested semantic edits remain recognizable. Because this analysis is qualitative, the examples do not establish comprehensive quantitative cross-model robustness.

**Table 1:** Quantitative comparison of prompt-related score, source-identity similarity, source-to-edit visual similarity, and deviation from the corresponding undefended edit. Results are reported as mean ± standard deviation. Arrows indicate the favorable direction of each metric (↑: higher; ↓: lower). Bold values indicate the best reference-edit-deviation result among the evaluated defense methods for the corresponding metric.

Method	CLIP-S ↑	FR ↓	CLIP-I ↑	PSNR ↑	SSIM ↑	LPIPS ↓
No Defense	0.090±0.056	0.620±0.287	0.814±0.162	–	–	–
PhotoGuard Encoder Attack	0.090±0.051	0.615±0.274	0.781±0.147	18.92±4.598	0.666±0.156	0.423±0.125
EditShield	0.083±0.054	0.633±0.271	0.816±0.144	21.58±5.493	0.721±0.159	0.317±0.148
Untargeted Encoder Attack	0.090±0.051	0.594±0.290	0.780±0.172	19.87±5.141	0.657±0.160	0.427±0.125
CW-$L_{2}$ Attack	0.098±0.044	0.516±0.306	0.720±0.181	10.51±2.571	0.527±0.165	0.450±0.150
VAE Attack	0.093±0.049	0.607±0.274	0.781±0.154	20.06±4.841	0.693±0.153	0.385±0.130
FaceLock	0.089±0.051	0.496±0.260	0.778±0.169	20.03±5.139	0.661±0.159	0.431±0.123
Anonymization-Guided Latent Perturbation (Ours)	0.089±0.051	0.567±0.265	0.784±0.155	22.93±5.310	0.747±0.143	0.278±0.137

**Table 2:** Threshold-based face-verification results on the common set of 432 source–edit pairs. The same thresholds, calibrated from cross-identity source–source impostor pairs, are applied to both methods. A downward arrow (↓) indicates that a lower value corresponds to lower source-identity similarity or lower same-identity acceptance.

Method	N	Mean FR ↓	TAR@FAR = 1% ↓	TAR@FAR = 0.1% ↓
Original HIVE	432	0.5590±0.3418	0.7060	0.5347
Ours	432	0.5219±0.3212	0.6829	0.4792

**Table 3:** Descriptive face-detection outputs across different methods.

Method	Average Confidence	Detection Count
No Defense (Clean)	0.6455	3813
PhotoGuard	0.6361	3958
EditShield	0.6537	3698
FaceLock	0.6339	3905
Ours	0.6447	3823

**Table 4:** Ablation study on auxiliary loss components. The default proposed method uses only $L_{anon}$; $L_{disrupt}$ and $L_{FR}$ are evaluated only as additional ablation terms. A checkmark indicates that the corresponding loss term is included, and a downward arrow (↓) indicates that a lower value is favorable.

$L_{anon}$	$L_{disrupt}$	$L_{FR}$	LPIPS ↓	FR ↓
✓			0.278±0.137	0.567±0.265
✓	✓		0.321±0.136	0.547±0.269
✓		✓	0.321±0.135	0.551±0.272
✓	✓	✓	0.322±0.135	0.547±0.271

**Table 5:** Control experiment using different latent-space optimization targets. Lower FR indicates lower source-identity similarity, while lower MSE and higher SSIM indicate smaller deviation from the corresponding undefended edit. Accordingly, ↓ denotes lower-is-better and ↑ denotes higher-is-better. Bold values indicate the best value for each metric among the evaluated target constructions.

Target	FR (Source, Edit) ↓	MSE vs. Original Edit ↓	SSIM vs. Original Edit ↑
No Defense	0.5269	–	–
Random Image	**0.4191**	0.0210	0.8183
Grayscale Image	0.4860	0.0101	0.9127
Source-Matched Anonymization (Ours)	0.4889	**0.0093**	**0.9196**

**Table 6:** FR cosine similarity for source, anonymized-target, and edited-image pairs. A downward arrow (↓) indicates that a lower value corresponds to lower identity similarity.

Pair	Mean FR ↓
Source ↔ Normal HIVE Edit	0.5269
Source ↔ Protected Edit	0.4889
Source ↔ Anonymized Target	0.3005
Anonymized Target ↔ Protected Edit	0.3230

**Table 7:** Identity similarity and perceptual distance on InstructPix2Pix. A downward arrow (↓) indicates that a lower value is favorable.

Method	FR ↓	LPIPS ↓
No Defense	0.610	–
PhotoGuard	0.543	0.428
EditShield	0.576	0.351
Encoder Attack	0.481	0.473
CW Attack	0.409	0.479
VAE Attack	0.615	0.390
FaceLock	0.244	0.477
Ours	0.544	**0.262**

**Table 8:** Ablation study on perturbation budget ϵ. A downward arrow (↓) indicates that a lower value is favorable.

ϵ	LPIPS ↓	FR ↓
0.01	0.203	0.610
0.02	0.278	0.567
0.03	0.469	0.535
0.04	0.349	0.505
0.05	0.370	0.487

**Table 9:** Robustness under practical image preprocessing on the same 800-image subset. FR is measured between the source image and the protected edited output. LPIPS, PSNR, and SSIM compare each protected edited output with the corresponding original undefended HIVE edit. The clean row contains no preprocessing. Arrows indicate the favorable direction of each metric (↑: higher; ↓: lower).

Preprocessing	FR ↓	LPIPS ↓	PSNR ↑	SSIM ↑	N
None (Clean)	0.4889±0.3092	0.2648±0.1180	23.56±5.32	0.9196±0.1167	800
Center crop 0.90	0.4824±0.3057	0.5034±0.0697	13.87±2.11	0.6584±0.1454	800
JPEG Q = 50	0.4854±0.3170	0.2827±0.1078	23.00±5.02	0.9168±0.1134	800
JPEG Q = 75	0.5010±0.3155	0.2527±0.1107	24.03±5.33	0.9297±0.1030	800
Median 3×3	0.5005±0.3203	0.2950±0.1193	22.56±5.34	0.9017±0.1296	800
Resize 0.75×	0.4890±0.3214	0.2761±0.1179	23.04±5.28	0.9120±0.1207	800

## Data Availability

The dataset used in this study is publicly available. The implementation of the proposed method is available at https://github.com/tiemhub/AnonymizerNoise (accessed on 19 September 2026).

## Abbreviations

The following abbreviations are used in this manuscript:

LDM	Latent Diffusion Model
VAE	Variational Autoencoder
FR	Face Recognition
LPIPS	Learned Perceptual Image Patch Similarity
PSNR	Peak Signal-to-Noise Ratio
SSIM	Structural Similarity Index Measure
CLIP	Contrastive Language–Image Pre-training
IP2P	InstructPix2Pix
